# Influence of peri-implantitis defect angles on morphology and the biocompatibility of the implant surfaces after Er, Cr: YSGG laser therapy: an ex vivo study

**DOI:** 10.1186/s12903-026-09278-3

**Published:** 2026-07-22

**Authors:** Alaa Hashim, Hadeel Gamal Almalahy, Nashwa El-Khazragy, Nevine H. Kheir El Din

**Affiliations:** 1 Department of Oral Medicine, Periodontology and Oral Diagnosis, Faculty of Dentistry, Ain Shams University, Cairo, Egypt; 2 Department of Clinical Pathology-Hematology and Ain Shams Medical Research Institute (MASRI), Faculty of Medicine, Ain Shams University, Cairo, Egypt; 3 Department of Burn, Plastic & Reconstructive Surgery, Skin Culture Lab, Al Nas Hospital, Cairo, 1376 Egypt

**Keywords:** Er, Cr: YSGG laser, Peri-implantitis, 3D-printed model, Infrabony defects, Peri-implant defect angle, Defect Morphology, Atomic force Microscopy, Biological response (biocompatibility)

## Abstract

**Purpose:**

The key obstacles in peri-implantitis therapy are the decontamination of the previously infected implant surface, surface integrity preservation, and enhanced biocompatibility. All are essential events for positive re-osseointegration. The principal hypothesis is that diverse peri-implantitis defect angles may affect the effectiveness of Er, Cr: YSGG laser therapy in a 3D-printed model of peri-implantitis. The primary objective of this study was to evaluate implant morphological changes through profilometer analysis with atomic force microscopy. The biological response (biocompatibility) assessment via the cell culture process to determine human osteoblasts (Saos-2) attachment, differentiation, and mineralization was the secondary objective.

**Materials and methods:**

50 mJ (1.5 W/30 Hz), 50% air, 40% water, and five cycles at five minutes were applied in the decontamination procedure with an Er, Cr: YSGG side-firing laser tip. This was applied to twenty-four tested implants. They were split into four test groups with peri-implant infrabony defect angles of 15°, 30°, 60°, and 90°. These defects were customized in a 3D-printed model. As controls, two native implants were examined.

**Results:**

There was no significant difference in implant surface roughness between the control and four tested groups. There was a slight reduction in implant surface roughness concerning 60-degree bone defect angulation. There was high alkaline phosphatase enzyme (ALP) activity, high osteoprotegerin (OPG) protein and gene expression, and the lowest soluble receptor activator of nuclear factor-kB ligand (RANKL) protein and gene expression in the 60-degree bone defect compared to other bone defect angulations.

**Conclusion:**

Er, Cr: YSGG laser therapy in the decontamination procedure of previously contaminated implants in peri-implantitis therapy is successful in preserving the implant surface integrity in diverse peri-implantitis defects without major surface roughness alterations; it shows favorable biocompatibility with an appropriate osteogenic response in the four studied groups. The 60-degree bone defect shows slightly enhanced surface roughness and osteogenic response. Defect angulation influences the surface roughness and cell response. The new pristine implant still had a superior cellular response compared to the previously decontaminated implant surface.

**Clinical relevance (scientific rationale for study):**

Peri-implantitis is a severe form of periodontal disease that affects the bone around dental implants. The primary challenge with this disease is cleaning the contaminated surface without causing further damage, allowing bone to grow, and maintaining the implant stability. In other words, the key issue for peri-implantitis treatment is maintaining a biocompatible implant surface during cleaning. If the implant surface is damaged, bone will not grow well. This study investigates the cleaning of an infected implant surface using the Er, Cr: YSGG laser, examines whether it maintains surface safety and functionality during decontamination of different bone defect shapes, and investigates the ability of osteoblasts to grow and attach to cleaned implants. Knowledge of how variations in bone defect shape affect cleaning outcomes and cellular responses will aid clinicians in selecting the most appropriate treatment modality for patients with peri-implantitis.

**Clinical trial registration:**

" Efficacy of Er: YSGG Laser in Treatment of Peri-implantitis". ClinicalTrials.gov ID: NCT05137821. First Posted date: 30 -11-2021

**Supplementary Information:**

The online version contains supplementary material available at https://doi.org/10.1186/s12903-026-09278-3.

## Introduction

The dilemma regarding the re-osseointegration of previously contaminated implant surfaces in peri-implantitis attracts researchers trying to find a suitable decontamination tool in the absence of a single well-defined therapeutic protocol [1]. The compromise of implant topography during the decontamination procedure was a crucial issue with questionable re-osseointegration, as it influences cell reaction and bacterial adhesion. Maintaining appropriate surface characteristics and biocompatibility allows adequate adhesion of cells to the surface, with subsequent growth [2, 3].

Peri-implantitis therapy is influenced significantly by the implant surface-cleaning technique. Additionally, defect angle and access geometry clearly influence performance [4–6]. Thus, obtaining reliable therapeutic approaches that evade unwanted surface alterations is mandatory. However, an ideal approach with predictable and favorable outcomes is still lacking. [7–9]. Consequently, persistent exploration of other recent approaches is needed to evade the drawbacks of conventional therapy. Er, Cr: YSGG laser device is one of the suggested approaches with valuable effects that need to be verified [10, 11].

Laser therapy has demonstrated sensitivity to parameters and techniques. All variables must be considered, including the duration of the laser application and the application distance [12, 13]. The tip angulation and distance relative to the implant surface are relatively significant in altering the implant surface damage patterns [14–16]. The laser irradiation angle modification significantly alters temperature distribution and tissue damage; higher angles lower the damage ratios [14]. Clinically, such an approach cannot be certain. Further research is necessary to address the restriction of the efficacy change with angulation discrepancies [17]. A significant knowledge gap exists regarding the effect of defect angulation on the decontamination capability of the Er, Cr: YSGG laser.

This study’s principal hypothesis is to evaluate the possible influence of diverse peri-implantitis defect angles on the effectiveness of Er, Cr: YSGG laser therapy in a 3D-printed model of peri-implantitis. The primary objective of this study was to evaluate implant morphological changes within diverse defect angles in a 3D-printed model of peri-implantitis following Er, Cr: YSGG laser therapy through profilometer analysis with atomic force microscopy. The biological response (biocompatibility) assessment via the cell culture process to determine human osteoblasts (Saos-2) attachment, differentiation, and mineralization was the secondary objective.

A PICO Question was implemented during this study (problem (various angle defects within a 3D-printed model of peri-implantitis), Intervention (Er: Cr: YSGG decontamination of previously contaminated implant surfaces), Comparative (native implants), Outcome (Surface roughness and cell response)) [18].

## Subjects and methods

### Overall study design

This investigation was structured as an ex vivo study, assessing twenty-six (26) implants^1^comprising twenty-four (24) experimental implants across four groups and two (2) new implants in the control group. Microsoft Excel randomly allocated the six implants in each group. The study protocol was approved by the Research Ethics Committee of the Faculty of Dentistry, Ain Shams University (ID: 210314). The study was conducted at the Laser Center, Ain Shams University, and registered at ClinicalTrials.gov (NCT05137821).

A custom CAD-designed, 3D-printed model (Exocad; AccuFab D1s, Shining3D, China) fabricated using resin material (Proshape Digital Solutions) was employed to simulate four standardized peri-implant infrabony defects, enabling efficient in vitro implant replacement and testing. [19].

The technology devised by Sahrmann was utilized to produce repeated peri-implant infrabony defects. Angulation was the sole variable that differed across these circumferentially standardized infrabony defects that were six millimeters deep. This was done with the intention of providing varying degrees of decontamination accessibility that were reflective of actual clinical situations [20].The variable defect angulations evaluated in this study were as follows: Group 1, 15°; Group 2, 30°; Group 3, 60°; and Group 4, 90°.

Three healthy volunteers were part of this research for the development of in-vivo biofilm plaque on the four examined groups of implants for four days (96 h); every volunteer used a rigid resin splint consisting of implants secured with an orthodontic wire to enable the natural accumulation of dental plaque [4].

**Fig. 1:**
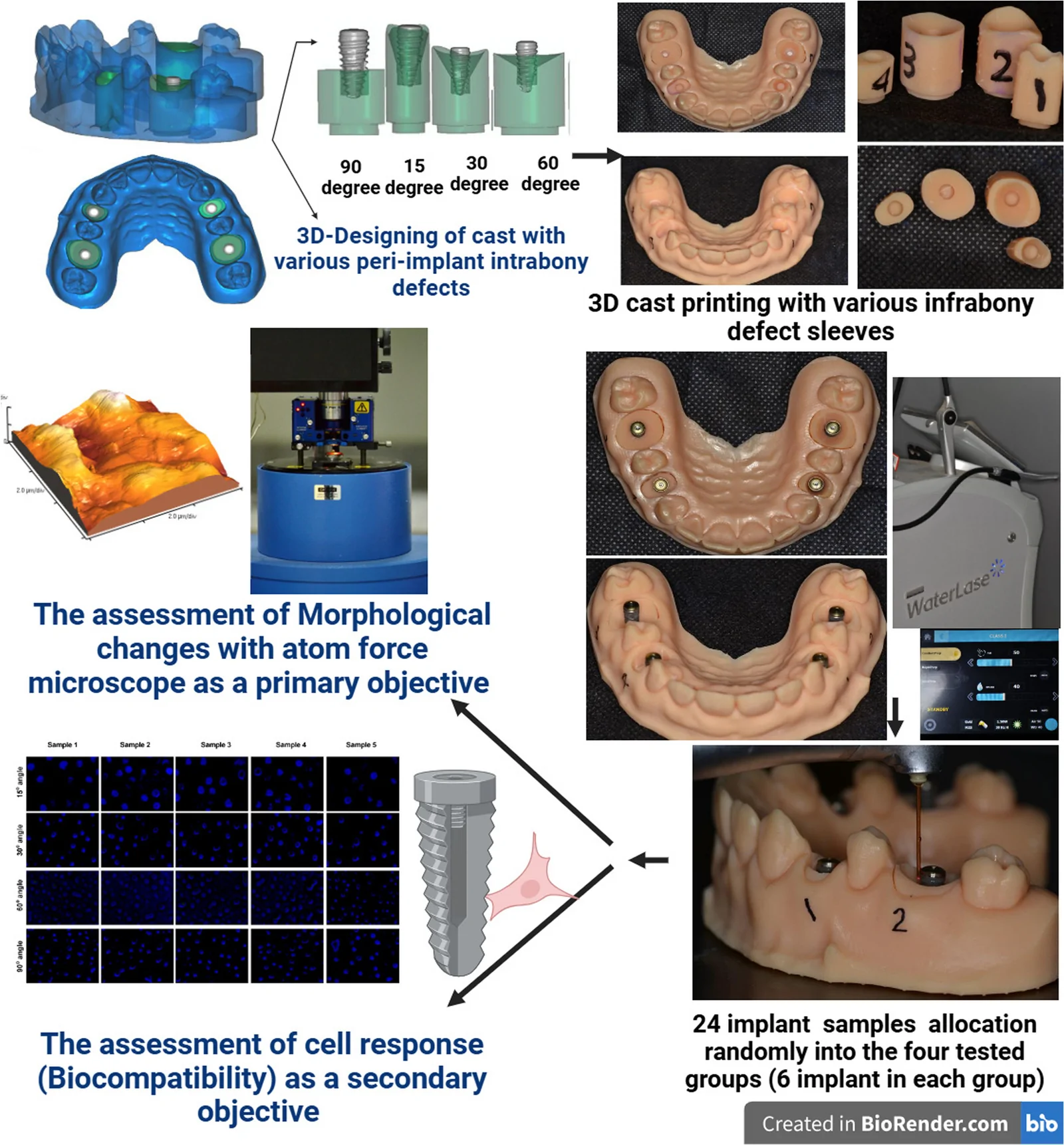
A summarized flow chart of the study protocol

Subsequently, in various peri-implant replicated bone defects, the 24 implants collected after biofilm accumulation were assessed for the impact of Er: Cr: YSGG laser decontamination therapy on both morphological changes and cell response. Two new implants were used for surface roughness and cell response analysis without biofilm accumulation. All implants were evaluated in the same ordered protocol. Biofilm accumulation and removal were done using the same protocol as in a previous study [4]. These steps are summarized in Fig. 1.

### Implant surface decontamination

50 mJ (1.5 W/30 Hz), 50% air, 40% water, and five cycles at five minutes were applied in the decontamination procedure with an Er, Cr: YSGG (Waterlase) 500-µm radial firing tip. This was applied in non-contact mode under standardized parameters, in circumferential apico-coronal cycles to ensure effective biofilm removal [4, 21–23]. This protocol was approved to have sufficient biofilm removal from the implant surface [4].

### Data collection

Morphological changes of the implant surface were determined in the study through profilometer analysis with an atomic force Microscopy (Silicon Nitride probe MLCT).

**Fig. 2:**
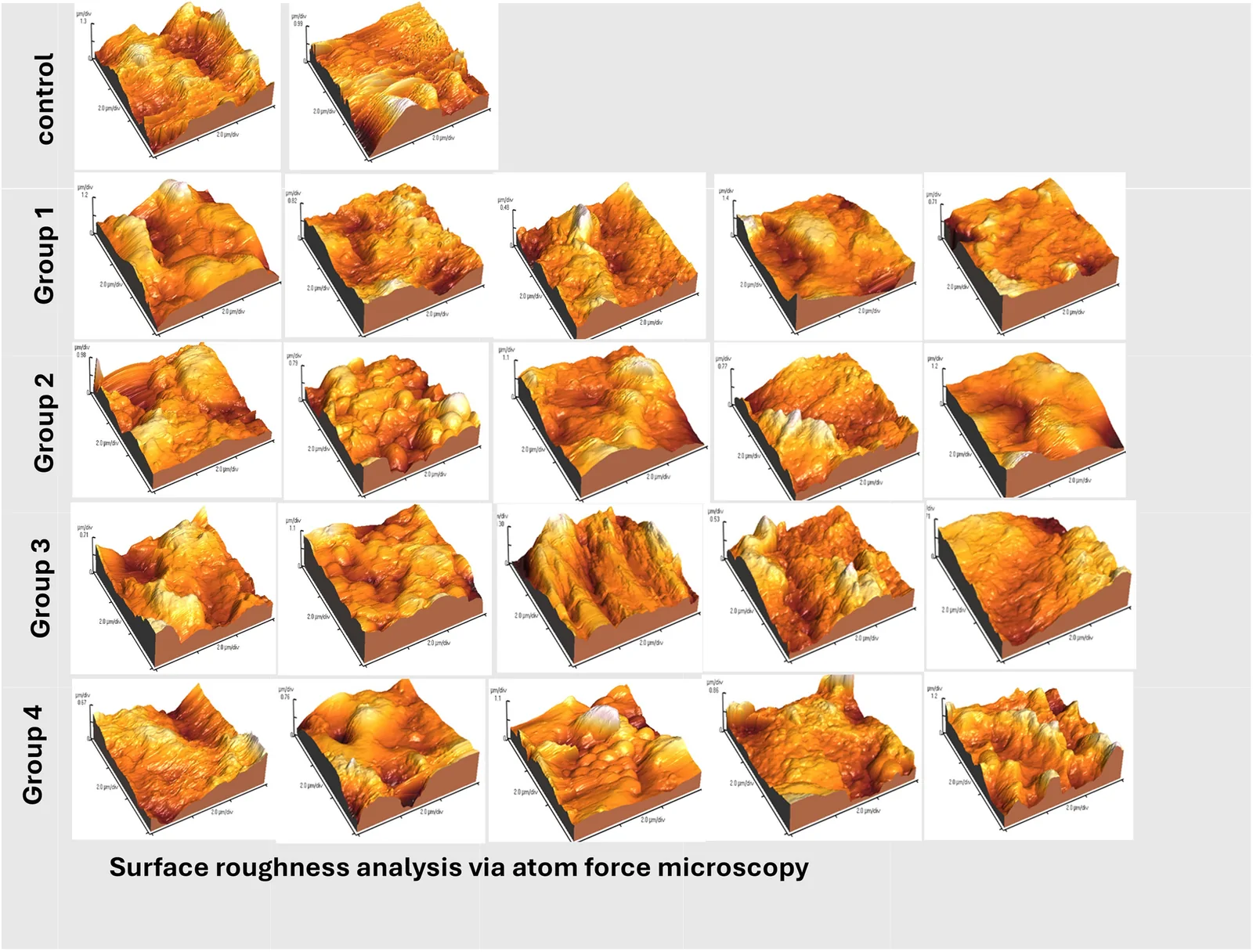
Surface roughness analysis via atomic force microscopy

A scan area of 6 × 6 µm2, with a scan rate of 1 Hz at Proscan 1.8 software, was used for controlling the scan parameters, with operating IP 2.1 software for image analysis (Fig. 2).

The average roughness is given by the average deviation of the data, referenced to the average of the data within the included areas. The average roughness is determined using the standard definition. In addition, the biological response (biocompatibility) of previously contaminated implant surfaces was evaluated after Er: YSGG laser therapy through a cell culture procedure to detect attachment, differentiation, and mineralization of human osteoblasts (Saos-2). This was assessed in the following ordered steps: osteoblastic cell proliferation estimation through an MTT assay, adhesion potential evaluation with the assessment of cell morphology under a fluorescence microscope, cell functional performance through Alkaline phosphatase enzyme (ALP) activity, osteogenic-related protein expression as OPG and RANKL proteins through ELISA, and osteogenic-related gene expression by qPCR.

First, the adherence of Saos-2 cells to the studied implants.

The Human Osteosarcoma cell line (Saos-2) was derived from a human primary osteosarcoma and used as osteoblast-like cells. Cells were maintained at 37 °C in 5% CO_2_ for 4 h for adherence. At the end of incubation, the non-adherent cells were washed with PBS; the adherent cells were fixed in 4% formaldehyde (Sigma-Aldrich, USA) and stained with 4′,6-diamidino-2-phenylindole (DAPI) (Invitrogen, Thermo Scientific, USA), and examined under a fluorescence microscope. Six separate implant samples (biological replicates). For each sample, three different fields were examined, averaged for each implant, and used as replicates for each biological sample (implant, *n* = 6). The number of cells/fields was used to represent the number of adherent cells/groups. Representative images show the adhered cells (blue) on the surfaces. Images were captured with a LABOMED Fluorescence microscope LX400, cat no: 9,126,000; USA. The magnification power is 40X, and the scale bar is 100 μm. No adhesion-promoting reagents were used.

Second, the ALP enzyme activity of cells on the studied implants. Saos-2 cells were introduced to plates with a density of 2.5 × 105 cells/ml DMEM supplemented with 29.2 mg/ml L-glutamine, 10,000 units/ml penicillin, 10 mg/ml streptomycin, and 10% fetal calf serum (FCS). ALP was measured in cell lysate using an enzyme-linked immunosorbent assay (ELISA), and its measurements were normalized to the cell count in each group.

Third, the expression of OPG and RANKL proteins in cells cultured on different dental implants to determine the differentiation of Saos-2 after being seeded on different implants. Cells were grown on the corresponding implants in a well culture plate for 7 days in a 37 °C and 5% CO2 incubator. At the end of incubation, the expression levels of Osteoprotegerin and sRANKL proteins were measured by ELISA.

Fourth, the expression of OPG and RANKL mRNA in cells cultured on different dental implants. To verify the osteogenic and osteoclastic changes of Saos-2 after being seeded on four different implants, cells were grown on the corresponding implants in a well culture plate for 7 days in a 37 °C and 5% CO2 incubator. At the end of incubation, the expression of OPG and RANKL genes was measured by quantitative real-time PCR. The gene expression was presented as fold change (FC) normalized to the control group.

### Statistical analysis

All collected data were statistically analyzed using a one-way analysis of variance test (ANOVA), comparing various peri-implantitis defect angulations and control groups.

All statistical analyses were performed using Graph Prism, version 12. Continuous data are presented as mean ± standard deviation. Normality and homogeneity of variance were assessed using the Shapiro–Wilk and Levene’s tests, respectively. A comparative analysis of the tested groups and the control regarding implant surface roughness (nm) was performed using the ANOVA test. Moreover, an ANOVA test, followed by Tukey’s multiple comparisons test, was conducted to find out the significant difference between the mean of the four tested groups versus the negative control group, considering all the tested parameters, including the number of adherent cells, ALP enzyme activity, osteogenic-related protein expression (OPG and RANKL) proteins, and their osteogenic-gene expression.

Power analysis indicated that eight implants (two per group) were sufficient to detect a large effect size (17.4) at 80% power and α = 0.05 [19]. A total of 26 implants, twenty-four (24) implants tested at four diverse infrabony defects that reflected human peri-implant defects in peri-implantitis, and two (2) control implants. Comparisons were conducted between different groups and the control group.

## Results

**Fig. 3:**
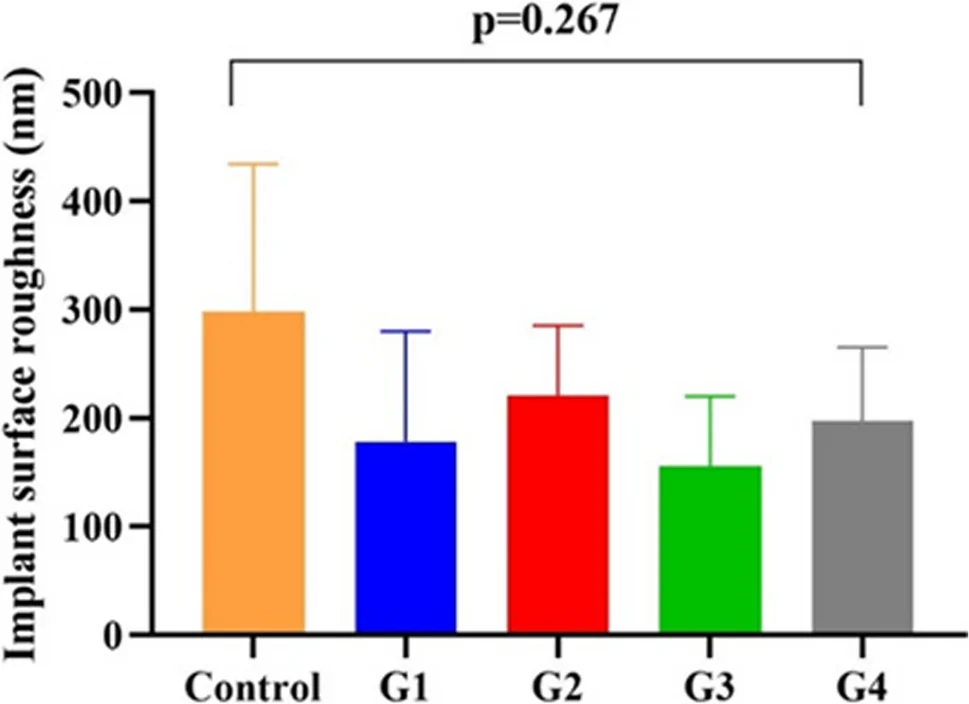
Bar chart showing the mean value of implant surface roughness (nm) in various groups compared to the control group. Group 3 (60°) showed a slight reduction in roughness among the tested groups

Initially, a one-way analysis of variance test (ANOVA) was conducted on the implant surface roughness (nm) of the analyzed atomic force microscopy images among the various tested peri-implantitis defect angulation groups and the control group. The results revealed no significant difference between the various tested groups compared to the control (*p* > 0.05); however, group 3 (60^o^ angulation of the replicated bone defect) (mean: 156) showed a slight reduction in the implant surface roughness compared to the control and other tested groups. The data are presented in Fig. 3.

Then, regarding surface biocompatibility, a comparative analysis of cell morphology and Saos-2 cell adherence to the tested implant surface was conducted. The number of cells/fields represented the number of adherent cells/groups. G3 (60^o^ bone defect angulation) showed the highest number of adherent cells among the tested groups (315 ± 10.1), compared with the negative control (366 ± 12.9).

**Fig. 4:**
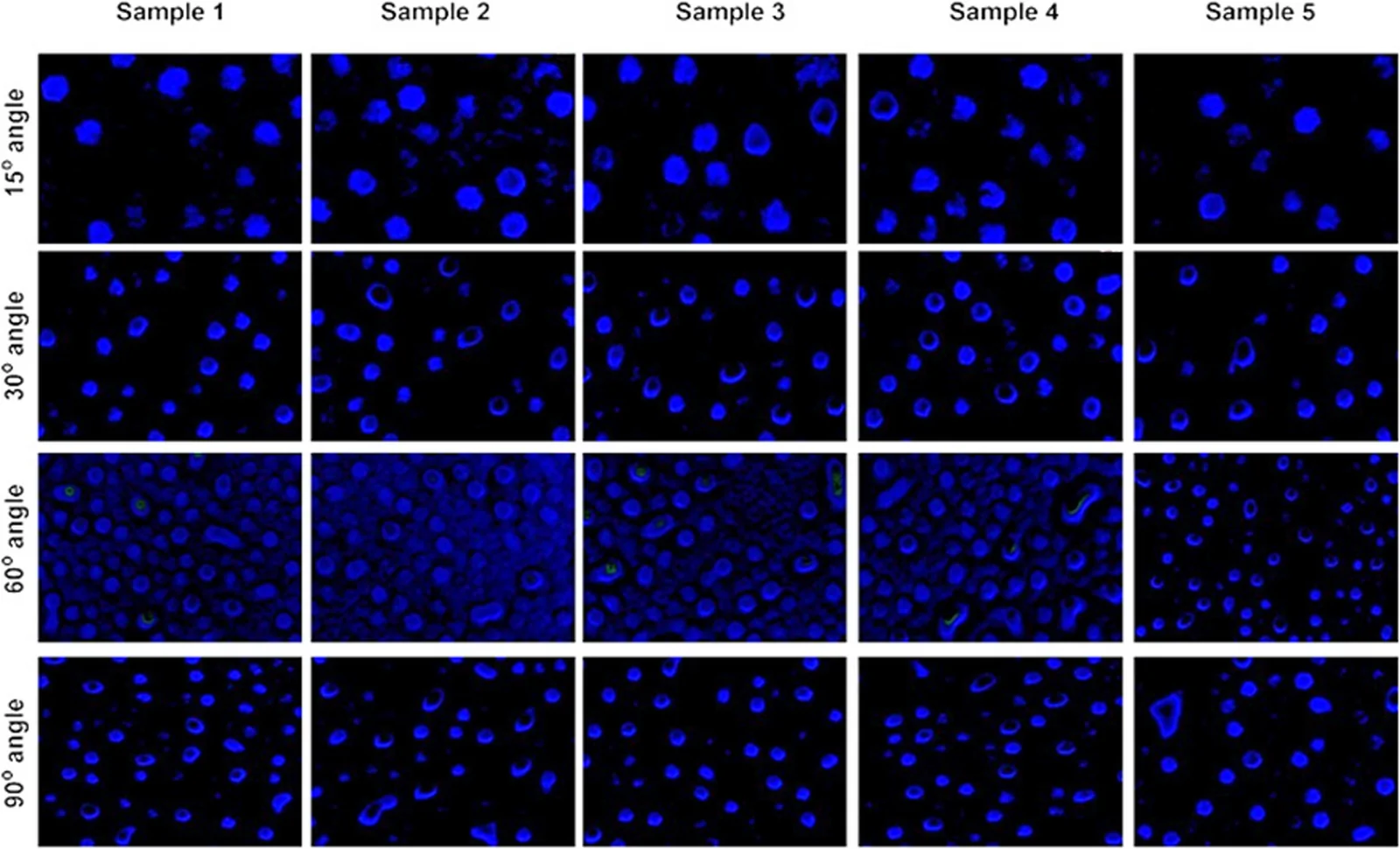
Osteoblast (Saos-2 cells) attachment onto dental implants under fluorescence microscopy. Representative images show the adhered cells (blue) on the surfaces, reflecting the cell morphology and cellular adhesion potential. The number of cells/fields was used to represent the number of adherent cells/groups

**Fig. 5:**
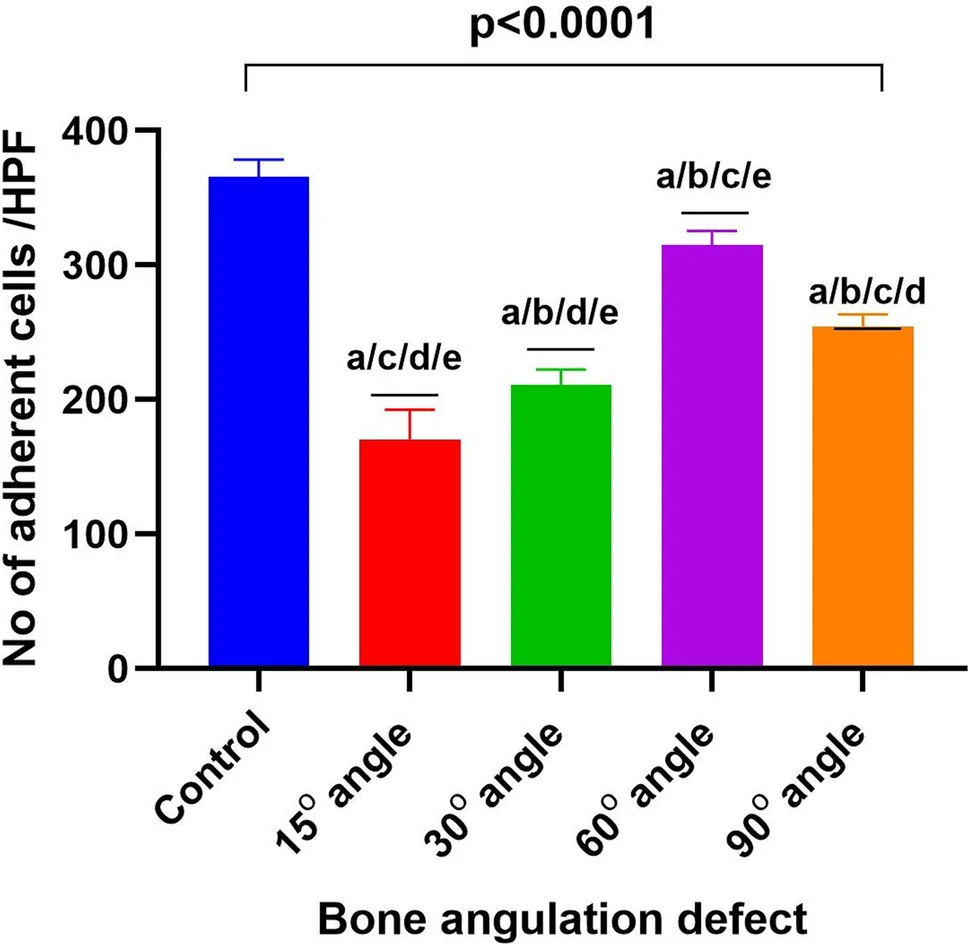
Bar chart demonstrating the number of adherent cells/HPF on various tested implant surfaces (p<0.0001)

However, G1(15° bone defect angulation) showed the least number of adherent cells (171 ± 22.1). The ANOVA test revealed a highly significant difference between each pair of the studied groups and the control group (*p* < 0.01). The Control group (new implant) still has the highest number of adherent cells when compared to other decontaminated implant groups with various defect angles. The results are presented in Table 1; Fig. 4, and Fig. 5.

**Table 1:** Descriptive analysis for the number of adherent Saos-2 cells on the dental implants/HPF^+^

Group	mean ± SD	Range	Statistics	
Control (*n* = 2)	366 ± 12.9	355–380		F: 125.9 *P* < 0.0001 [HS]
G1 (15^o^) (*n* = 6)	171 ± 22.1	150–200		
G2 (30^o^) (*n* = 6)	211 ± 11.4	200–230		
G3 (60^o^) (*n* = 6)	315 ± 10.1	300–325		
G4 (90^o^) (*n* = 6)	254 ± 9.62	240–265		

^+^*HPF* High power field (100x)

In addition, a comparative analysis of Saos-2 cells’ functional performance through ALP enzyme activity was conducted. It was detected using an enzyme-linked immunosorbent assay (ELISA).

**Fig. 6:**
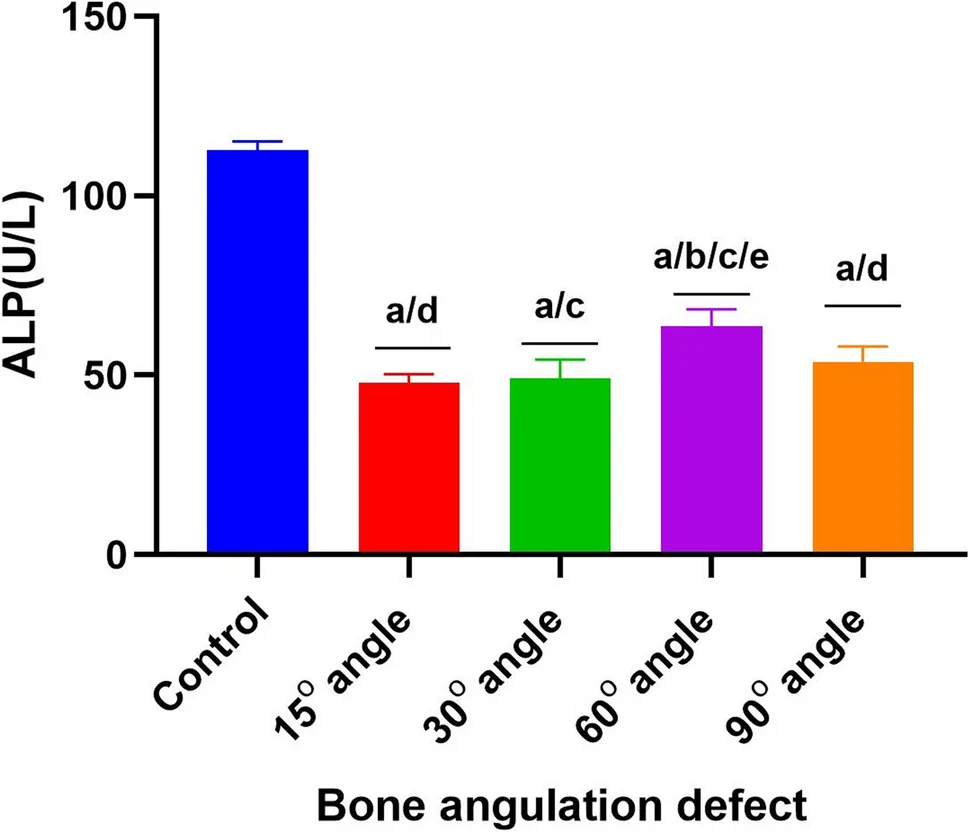
Bar chart demonstrating the ALP activity (U/L) in Saos-2 cells in different implant groups (p<0.0001). The highest ALP activity was associated with cells adherent to the 60° bone defect angulation group among the tested groups. The least ALP activity was associated with cells adherent to the 15° bone defect angulation group

The results revealed a highly significant difference between the ALP activity in cells adherent to various implant groups (*p* < 0.0001). The highest ALP activity was associated with cells adherent to the 60° bone defect angulation group among the tested groups (63.7 ± 4.65); however, the cells in the 15° bone defect angulation group showed the lowest ALP activity (47.8 ± 2.6). Multiple comparisons analysis showed a highly significant difference between various groups (*p* < 0.05), compared to the control group. The Control (new implant) group still has the highest ALP activity (113 ± 2.3), when compared to other decontaminated implant groups with various defect angles. The data are presented in Table 2 and Fig. 6.

**Table 2:** Descriptive analysis of the alkaline phosphatase activity (U/mL) in Saos-2 cells on the dental implants/HPF

Group	mean ± SD	Range	Statistics
Control (*n* = 2)	113 ± 2.3	111–115	F: 144.4 *P* < 0.0001 [HS]
G1 (15^o^) (*n* = 6)	47.8 ± 2.6	43.6–50.2	
G2 (30^o^) (*n* = 6)	49.1 ± 5.36	42.9–56.5	
G3 (60^o^) (*n* = 6)	63.7 ± 4.65	58.5–70.6	
G4 (90^o^) (*n* = 6)	53.8 ± 4.22	48.6–59.6	

Moreover, comparative analysis of osteogenic-related protein expressions, such as OPG and RANKL proteins through (ELISA), and their osteogenic-related gene expression by quantitative Real-time (qPCR) in adherent Saos-2 cells of various groups was performed.

The obtained results showed a highly significant difference regarding osteogenic-related protein expressions, such as (OPG) and (RANKL) proteins, in cells adherent to various implant groups (*p* < 0.0001).

**Fig. 7:**
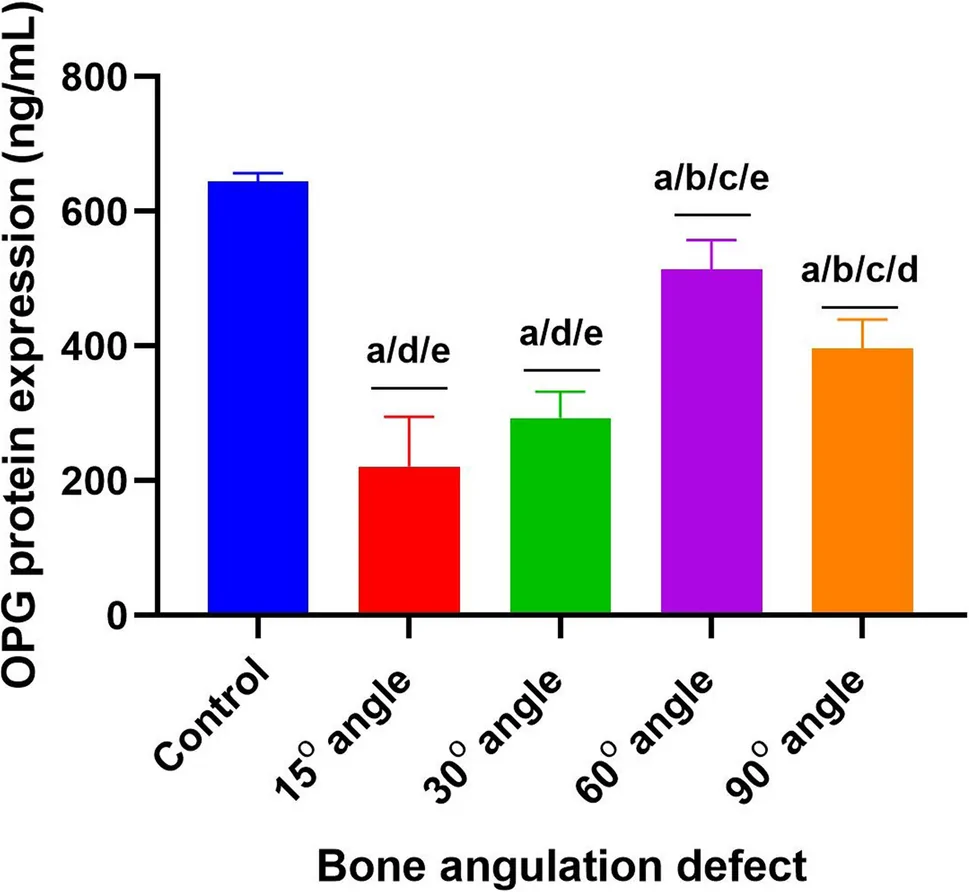
Bar chart illustrating the OPG protein expression (ng/mL) in Saos-2 cells in various implant groups (p<0.0001). The cells in group 3 (60° bone defect angulation) showed the highest OPG protein expression among the tested groups. The cells in group 1 (15° bone defect angulation) showed the Lowest expression of the OPG protein

**Fig. 8:**
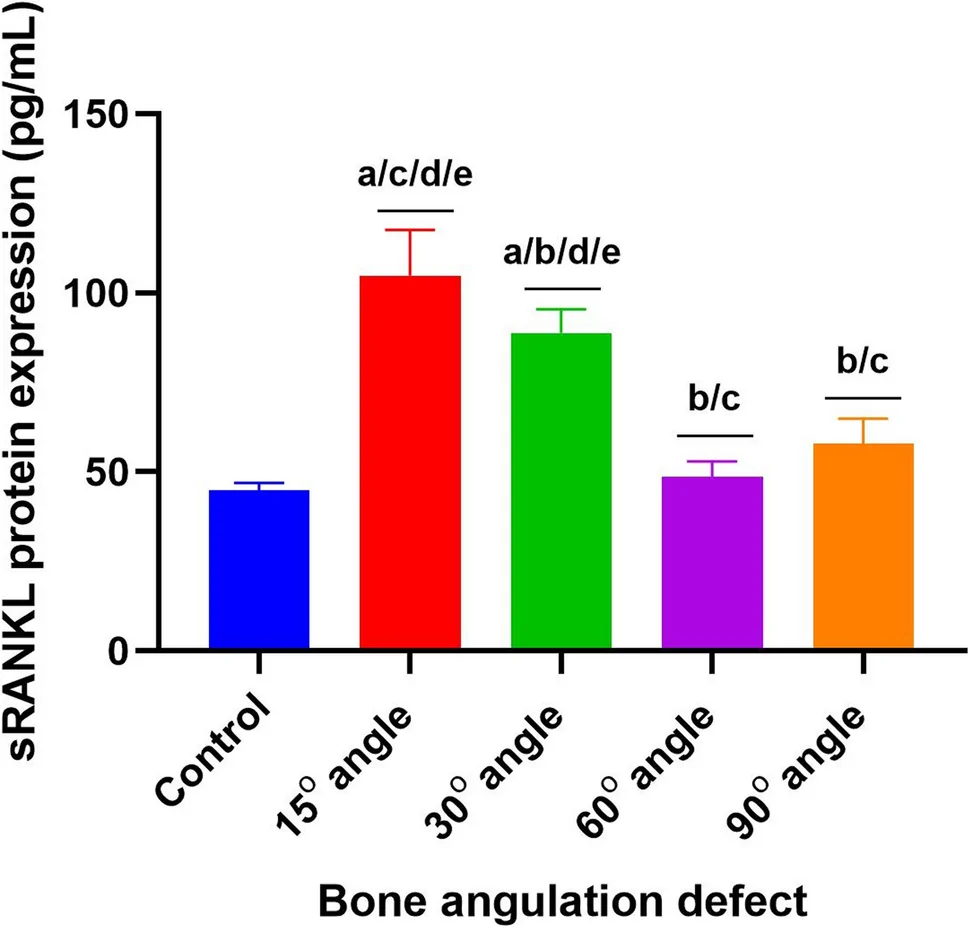
Bar chart illustrating the sRANKL protein expression (ng/mL) in Saos-2 cells in various implant groups (p<0.0001). The cells in group 3 (60° bone defect angulation) showed the Lowest expression of sRANKL protein among the tested groups. The cells in group 1 (15° bone defect angulation) showed the highest expression of the sRANKL protein

The cells in group 3 (60° bone defect angulation) showed the highest expression of OPG protein among the tested groups (514 ± 43.6), while this group showed the least expression of RANKL protein (48.6 ± 4.26). However, the cells in group 1 (15° bone defect angulation) showed the least expression of OPG protein (221 ± 73.9), with the highest expression of RANKL protein (105 ± 12.8). The Control (new implant) group still has the highest OPG protein expression and the lowest RANKL protein expression, when compared to other decontaminated implant groups with various defect angles. The data are presented in Figs. 7 and 8.

Similarly, the expression of both OPG and RANKL genes in the studied groups showed highly significant differences in cells adherent to the various implant groups (*p* < 0.0001). The gene expression was presented as fold change (FC) normalized to the control group.

**Fig. 9:**
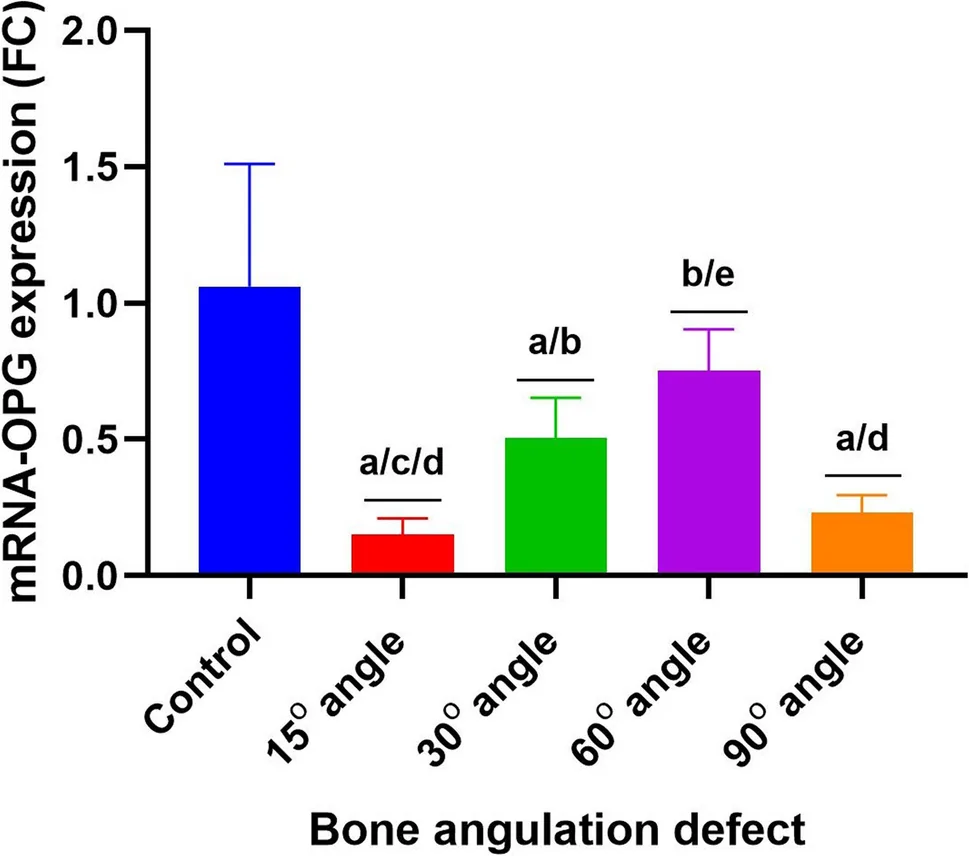
Bar chart illustrating the OPG gene expression (FC) in Saos-2 cells in different dental implants (p<0.0001). The cells in group 3 (60° bone defect angulation) showed the highest OPG gene expression among the tested groups. The cells in group 1 (15° bone defect angulation) showed the Lowest expression of the OPG gene

**Fig. 10:**
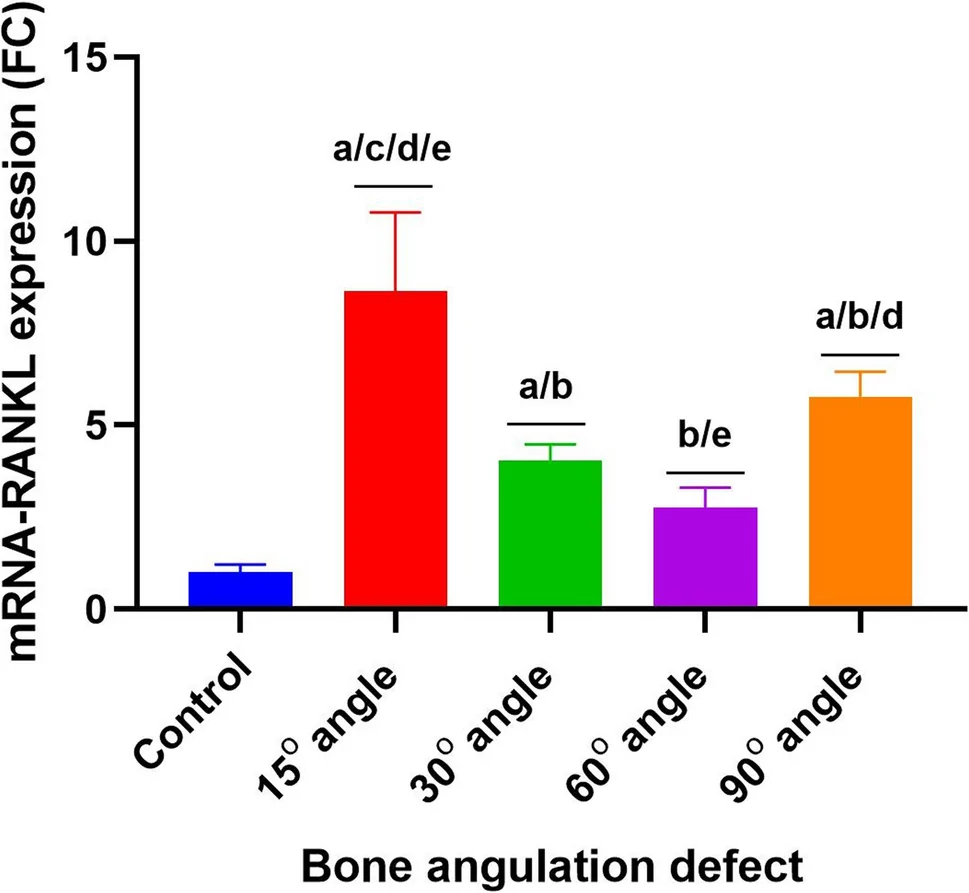
Bar chart illustrating the RANKL gene expression (FC) in Saos-2 cells in different dental implants (p<0.0001). The cells in group 3 (60° bone defect angulation) showed the Lowest expression of the sRANKL gene among the tested groups. The cells in group 1 (15° bone defect angulation) showed the highest expression of the sRANKL gene

Among the tested groups, the cells in group 3 (60^o^ bone defect angulation) showed the highest expression of OPG genes (0.752 ± 0.152), while this group showed the least expression of RANKL genes (2.77 ± 0.53). However, the cells in group 1 (15^o^ bone defect angulation) showed the least expression of OPG genes (0.152 ± 0.06) with the highest expression of RANKL genes (8.65 ± 2.14). The Control (new implant) group still has the highest OPG gene expression and the lowest RANKL gene expression, compared with other decontaminated implant groups with various defect angles. The data are presented in Figs. 9 and 10.

## Discussion

The selected laser parameters linked with the delivery system properties are crucial in the process of picking among several devices, as both impact the spatial energy distribution and power density transferred to the implant surface [13]. Re-osseointegration can be achieved around adequately decontaminated implants through direct structural and functional bone–implant integration [24].

There are multiple demands in the selected therapeutic protocol of peri-implantitis, such as the successful decontamination of previously contaminated implant surfaces, which has been proven before in another article with the same methodological protocol [4].

Owing to the lack of knowledge regarding linking defect angulation with both surface roughness and osteoblastic response. Further research investigating the impact of defect shape on reconstructive and resective outcomes is recommended [25].

This study hypothesizes that diverse peri-implantitis defect angles may affect the effectiveness of Er, Cr: YSGG laser therapy in a 3D-printed model of peri-implantitis. They were allocated into four tested groups with peri-implant infrabony defect angles of 15°, 30°, 60°, and 90°. The primary objective of this study was to evaluate implant morphological changes through profilometer analysis with atomic force microscopy. The biological response (biocompatibility) assessment via the cell culture process to determine human osteoblasts (Saos-2) attachment, differentiation, and mineralization was the secondary objective. However, clinical implications and re-osseointegration-related conclusions should be stated more cautiously, as Saos-2 cells do not fully represent primary osteoblasts or mesenchymal stem cells. Additionally, contaminated, untreated, and conventionally treated control groups were not included.

### Morphological changes

There was a non-significant reduction in the surface roughness when compared to the control group. Therefore, this laser device with the reported settings was verified to be safe in diverse peri-implantitis defect angles. This safety was verified in the literature [26, 27].

The angle variation is claimed to influence the surface roughness with subsequent influence on cell biocompatibility. The influence of peri-implantitis defect angle on the implant surface roughness in peri-implantitis therapy with Er, Cr: YSGG laser is not directly verified in the literature. However, several studies show how Er, Cr: YSGG laser parameters (power, pulse, spray, tip geometry) and delivery settings (application angle and distance) affect energy density with a suspected influence on surface roughness. Therefore, Er, Cr: YSGG outcomes depend strongly on how energy is delivered; the defect angle itself, which is suspected to modulate the application angle, was not directly tested; any change that concentrates or spreads the beam over an area will likely alter both effective energy density and resulting surface roughness [13].

There was a noticeable surface roughness reduction in group 3 (60° bone defect angulation). This is attributed to the alteration in application angle that impacts laser energy dispersion, as any target fluctuations influence the laser–target interaction [13]. Similarly, Cr: YSGG therapy has demonstrated a modification in the Ti surface properties with enhanced biological response of the treated surfaces, besides limited bacterial adhesion [28, 29].

However, other studies showed surface alterations with melting, flattening, or cracking. This is suspected to be a defective operation mode or a defective laser parameter selection [30].

The selected tool for surface alteration detection, Atomic Force Microscopy (AFM), satisfies all the requirements by characterizing the specimen’s surface morphology, mechanical properties, and atomic-scale resolution through a 3D image that allows description of surface organization as well as surface roughness (Ra) of titanium surfaces. Additionally, it is preferred to conduct imaging near physiological contexts using high-resolution images that include surface height information to enhance biological relevance [31, 32].

### Surface biocompatibility in terms of cell proliferation and adherence

G3 (315 ± 10.1) showed the highest number of adherent cells /HPF compared to the negative control (366 ± 12.9), followed by G4 and G2. However, G1 showed the minimum number of adherent cells. These results revealed the relationship between the adherence of Saos-2 cells and the angle of the bone defect.

The 60° bone defect angulation had the best adherence of cells among the tested groups. A strong association between the adherence of Saos-2 cells and the angle of the bone defect was observed. However, the control new implant still has better cell adherence compared with the decontaminated implant surface. This indicates that the new pristine implant still has better biocompatibility compared to previous decontaminated surfaces.

The superior cell adherence in G3 could be verified by the superior reduction in the surface roughness, with proven accessibility to the defect in the decontamination therapy. However, G1 showed the minimum adherent cells because of the defective angle tightening, with less evident accessibility, which allowed only a mild reduction in the % of implant surface area/biofilm surface area ratio compared to other groups, as discussed before [4].

This variation in adherence was assumed to be a mirror of surface roughness changes with influence on cell viability. The surface roughness alterations, such as surface damage, were not the only circumstances with a negative impact on the biological response. Additionally, the alteration of other surface characteristics, such as surface energy, is supposed to have an impact [33].

Residual plaque biofilm areas are suspected to influence the viability of (Saos-2) cells with reduced tissue integration by bacterial contamination of a titanium surface. There is a postulated alteration in the surface characteristics through assumed influence on its dioxide layer, resulting in a lower surface [4, 34].

These outcomes are consistent with other studies that proposed that Er, Cr: YSGG laser has a negligible effect on the surface, with preserved osteoblastic response through the ability to absorb osteogenic cells for further division and differentiation [6, 35].

In contrast, other findings postulated that the original biocompatibility of pristine titanium surface is incapable of being restored with any suggested decontamination devices, including Er, Cr: YSGG laser [9].

MTT assay used in this study, which is the most popular test regarding viability detection, allows the detection of any small alteration in cell metabolic activity, so it is a sensitive modality [3, 36].

### Osteogenic ALP activity

There was a highly significant difference in ALP activity in cells adherent to different dental implants. The maximum ALP activity was shown in cells adherent to the 60° bone defect angulation group. On the contrary, the lowest ALP activity was shown in the 15° bone defect angulation group. The control group ( new implant ) still had a superior ALP activity compared to the previously decontaminated implant surface.

Therefore, the Saos-2 cells’ ALP activity was verified to be related to the peri-implant defect angulation. This boosted cellular function performance could be a consequence of the enhanced biological response in the 60° bone defect angulation group. Unfortunately, no comparable studies have been done to compare the outcomes.

### Osteogenic protein and gene expression

G3 (60° bone defect angulation) showed a higher OPG protein and gene expression, while showing the lowest RANKL protein and gene expression, representing a good cellular response. The highest RANKL protein and gene expression in G1, with the lowest OPG protein and gene expression, indicates an elevation of the osteoclastic response (poor cellular response).

These results revealed a superior osteogenic (osteoblastic) response in G3, with the least osteogenic response in G1. Therefore, the difference in peri-implant defect angle was demonstrated to impact the osteoblastic response of the cells when the Er, Cr: YSGG decontamination therapy was accomplished in peri-implantitis therapy, besides its proven influence on the decontamination efficacy [4].

The control group ( new implant ) still had superior OPG protein and gene expression, and the lowest RANKL protein and gene expression compared to the previously decontaminated implant surface.

These results were parallel to the previous outcomes, as superior adherence, boosted ALP activity, and raised OPG protein and gene expression, with the lowest RANKL protein and gene expression in G3 (60° bone defect angulation group). Likewise, minimal ALP activity, lowest OPG protein and gene expression, with the highest RANKL protein and gene expression, and the least combined number of adherent cells in G1 (15° bone defect angulation group).

Osteoprotegerin and RANKL protein expressions were analyzed by the ELISA technique. However, their osteogenic-related gene expressions were analyzed by quantitative Real-time PCR. The selection of these tests was due to their accuracy in determining the in-vitro osteoblastic response successfully in multiple previous studies [37, 38]. However, no similar research has been conducted to compare these results.

Regarding the limitations and strengths, this research’s limitations resembled the general drawbacks of ex-vivo studies; these drawbacks are dependent on the gap between laboratory studies and clinically expected aspects. The reduction of this gap was expected to enhance the relation between the findings and clinical reality with the availability of their generalization in applied clinical therapy [39]. Clinically challenging peri-implant defects with unlimited variations in depth, angulation, and width may impact the effectiveness of decontamination and cellular response [4, 20]. The possibility of surface re-infection through saliva or blood, and the access limitations in clinical situations, such as the tongue, cheek, the presence of suprastructure, and the adapted gingival tissue. All these factors influence the quality of the decontamination process and the angulation of the laser tip application. The suspected influence on the efficacy of Er, Cr: YSGG laser is because of its sensitivity to water, which is abundant in gingival fluid, saliva, and blood [40]. Additionally, variable clinical patient-related factors. Future studies with a larger sample size, incorporating contaminated untreated and conventionally treated controls, are recommended. The use of Saos-2 osteoblast-like tumor cells limits the direct extrapolation of the findings to primary osteoblast behavior and clinical re-osseointegration.

The strength of this study was the simulation of the real clinical conditions through using a commercially available screw-shaped implant rather than other discs and cylinders, the development of a natural in-vivo developed biofilm rather than another highly adherent ink materials or in-vitro developed biofilm to avoid differences in adhesion force or biofilm complexity, and this study was designed to generate four experimentally created defects with various angulations resemble some natural challenges in clinical situations. The influence of defect angulation on surface roughness and cell response (biocompatibility) on the decontamination efficacy of Er, Cr: YSGG laser in peri-implantitis therapy remains poorly understood and has not been adequately studied in the literature.

Er, Cr: YSGG laser decontamination of previously infected implant surfaces across varying 3D-printed peri-implantitis defect angulations appeared biocompatible in this ex vivo model. The 60-degree defect showed enhanced performance due to the triple superiority in decontamination effectiveness, as proved before [4], surface roughness reduction, and superior osteogenic response.

Therefore, these findings suggest the potential to support re-osseointegration of previously contaminated implants following Er, Cr: YSGG laser decontamination by assessing surface morphology via atomic force microscopy and osteoblast (Saos-2) biological responses, assuming preserved surface integrity and biocompatibility.

However, the new pristine implant still has better biocompatibility compared to previously decontaminated surfaces due to the superiority of cellular response in terms of superior cellular adherence, ALP activity, the highest OPG protein and gene expression, and the lowest RANKL protein and gene expression.

## Conclusion

This Er, Cr: YSGG laser device with the stated settings demonstrated a favorable safety profile under the experimental conditions in diverse peri-implant defects with a non-significant reduction in the surface roughness that enhances cellular biocompatibility.

The angle of bone defect modulates the osteoblast proliferation, adherence, morphology, protein expression, and gene expression of (Saos-2) cells.

A superior osteogenic (osteoblastic) response in G3(60° bone defect angulation), with the least osteogenic response in G1 (15° bone defect angulation group).

Superior adherence, boosted ALP activity, raised OPG protein and gene expression, with the lowest RANKL protein and gene expression, were presented in G3 (60° bone defect angulation group). Likewise, minimal ALP activity, lowest OPG protein and gene expression, with the highest RANKL protein and gene expression, and the least combined number of adherent cells in G1 (15° bone defect angulation group).

The new pristine implant still had a superior cellular response compared to the previously decontaminated implant surface. A caution regarding (Saos-2) cells’ behavior and control group determination should be considered in other studies.

## Supplementary Information

Supplementary Material 1.

## Data Availability

All data created or analyzed during this study are incorporated in this published article [and its original data to declare will be provided if required by the reviewers].

## Footnotes

1 (machined implants; 3.5 mm diameter, 8 mm length).

## Abbreviations

ALP: High Alkaline Phosphatase Enzyme
OPG: Osteoprotegerin
CAD: Computer-Aided Design
RANKL: Receptor Activator of Nuclear Factor Kappa-B Ligand
ELISA: Enzyme-Linked Immunosorbent Assay
AFM: Atomic Force Microscopy
Er, Cr: YSGG: Erbium, Chromium-doped Yttrium, Scandium, Gallium, Garnet
