# Supplementary material for: Influence of peri-implantitis defect angles on morphology and the biocompatibility of the implant surfaces after Er, Cr: YSGG laser therapy: an ex vivo study
Source: BMC Oral Health. 2026 Jul 22;26:1673. doi: 10.1186/s12903-026-09278-3 (PMC13548605; doi:10.1186/s12903-026-09278-3)
Supplement: Supplementary file 1 [file 12903_2026_9278_MOESM1_ESM.pdf]

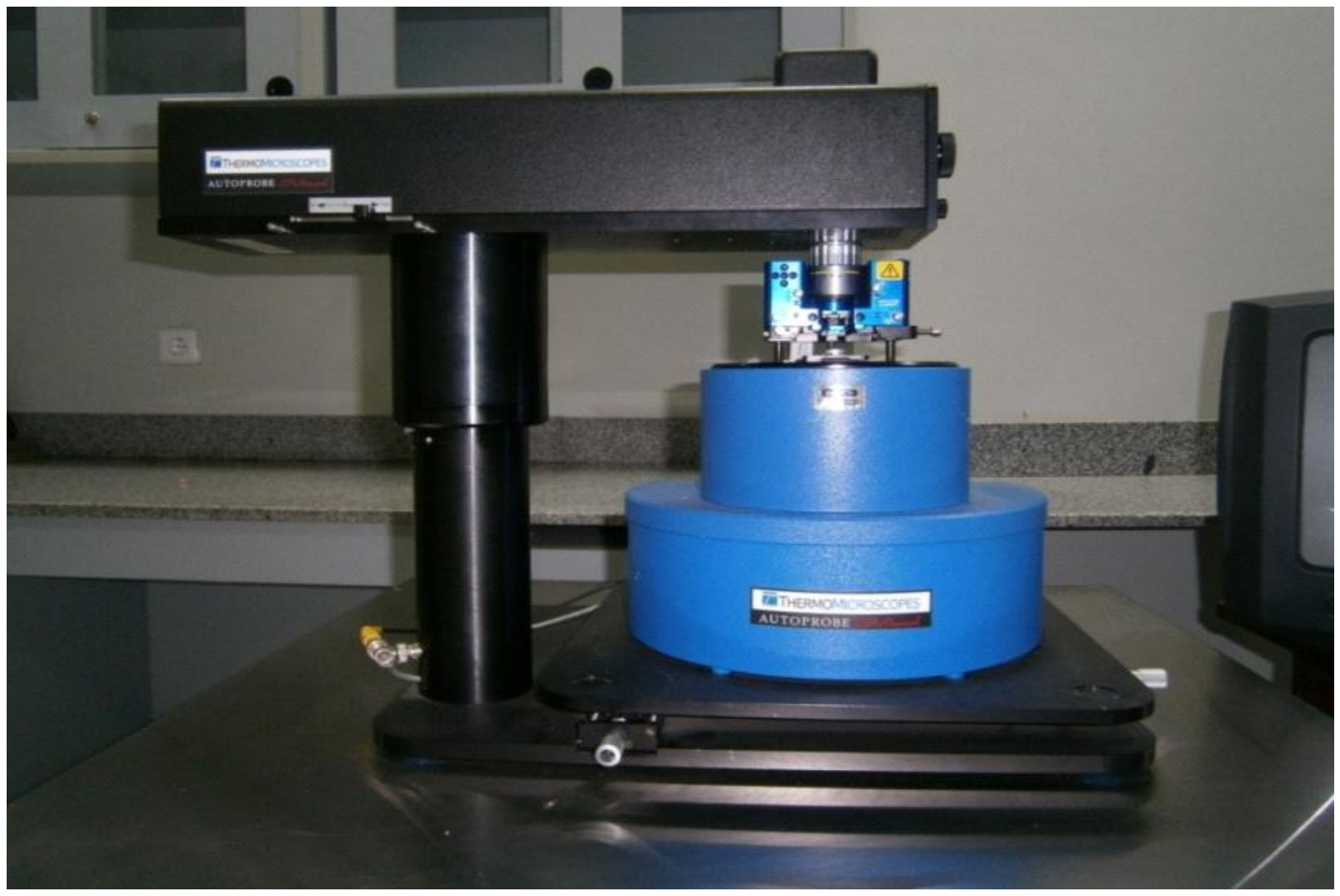

The roughness of the samples is measured using Atomic force Microscope Autoprobe cp-research head manufactured by:- Thermomicroscope operated in contact mode using Silicon Nitride probe model MLCT manufactured by Bruker. Scan area 6x 6 μm^2, scan rate 1 Hz . proscan 1.8 software was used for controlling the scan parameters and IP 2.1 software for image analysis.

Surface parameters

- Rp-v (Maximum peak-to-valley distance)
- Rms rough (root-mean-squared roughness)
- Ave rough (average roughness)
- Mean height (mean height)
- Median height (median height)
- Volume
- Peak
- Valley
- Surface Area
- Projected Area
- Bearing Ratio at two levels.

Ave roughness: The average roughness is given by the average deviation of the data, referenced to the average of the data within the included areas. The average roughness is determined using the standard definition:

$$R_{ave} = \sum_{n=1}^{N} \frac{|z_n - \bar{z}|}{N}, \text{ where } \bar{z} = \text{mean z height}$$

In Region Analysis mode, the average roughness refers only to the included areas defined by a region group, with N given by the number of data points in the included areas. The measurement is independent of the marker settings.

Rms roughness: The rms roughness (root-mean-squared roughness) is given by the standard deviation of the data, determined using the standard definition:

$$R_{rms} = \sqrt{\frac{\sum_{n=1}^{N} (z_n - \bar{z})^2}{N - 1}}, \text{ where } \bar{z} = \text{mean z height}$$

Mean height: The mean height is given by the average height within the included areas defined by a region group.

$$\bar{z} = \frac{1}{N}\sum_{n=1}^{N} z_n, \text{ where } \bar{z} = \text{mean z height}$$

where N is the number of data points within the area defined by the region group.

Median height: The median height is the height value which divides the height histogram into two equal areas. At the median value, 50% of the data points have higher values and 50% have lower values. The median height gives the same result as bearing ratio level 50%.

Volume: The volume measurement gives the volume underneath the included areas defined by a region group. The zero reference is the minimum data point of the entire image.

Rp-v: The Rp-v measurement gives the maximum peak-to-valley distance within the included areas defined by a region group. Rp-v is given by the highest data point in an area defined by a region group minus the lowest point:

$$R_{p\text{-}v} = z_{max} - z_{min}$$

Surface area: The surface area measurement gives an approximation of the surface area within the included areas defined by a region group. The surface area corresponding to a data point is approximated by doubling the area of the triangle connecting it to two of its nearest neighbors. The resulting rectangular areas corresponding to each data point are added.
